# *Agrobacterium rhizogenes*—mediated transformation of *Pisum sativum* L. roots as a tool for studying the mycorrhizal and root nodule symbioses

**DOI:** 10.7717/peerj.6552

**Published:** 2019-03-06

**Authors:** Irina V. Leppyanen, Anna N. Kirienko, Elena A. Dolgikh

**Affiliations:** All-Russia Research Institute for Agricultural Microbiology, Saint-Petersburg, Russia

**Keywords:** *Agrobacterium rhizogenes*, Composite plants, Mycorrhizae, Pea transformation, Nodulation

## Abstract

In this study, we demonstrated the successful transformation of two pea (*Pisum sativum* L.) cultivars using *Agrobacterium rhizogenes*, whereby transgenic roots in the resulting composite plants showed expression of the gene encoding the green fluorescent protein. Subsequent to infection with *A. rhizogenes*, approximately 70%–80% of pea seedlings developed transgenic hairy roots. We found out that the transgenic roots can be efficiently nodulated by *Rhizobium leguminosarum* bv. *viciae* and infected by the arbuscular mycorrhizal (AM) fungus *Rhizophagus irregularis*. The morphology of nodules in the transgenic roots was found to be identical to that of nodules observed in wild-type roots, and we also observed the effective induction of markers typical of the symbiotic association with AM fungi. The convenient protocol for highly efficient *A. rhizogenes*-mediated transformation developed in this study would be a rapid and effective tool for investigating those genes involved in the development of the two types of symbioses found in pea plants.

## Introduction

Pea (*Pisum sativum* L.) is one of the most agriculturally important legumes and is a potential target for crop improvement. In addition, large collections of pea mutants are available for the elucidation of gene function in the regulation of many processes in this plant (Jacobsen & Feenstra, 1984; Postma, Jacobsen & Feenstra, 1988; Kneen, Weeden & Larue, 1994; Borisov et al., 2007; Yang et al., 2017). The experimental approaches for such analyses are mainly based on complementation tests for mutants, as well as ectopic expression of transgenes or gene silencing mediated via the expression of hairpin RNA. However, in comparison with other legumes such as *Lotus japonicus* and *Medicago truncatula* (Schauser et al., 1999; Ané et al., 2002; Limpens et al., 2003; Madsen et al., 2003; Arrighi, 2006; Gonzalez-Rizzo, Crespi & Frugier, 2006; Heckmann et al., 2006; Tirichine et al., 2006; Smit et al., 2007; Floss et al., 2013; Yano et al., 2017), the technique of transformation necessary for such experiments, as well as for crop improvement, has not been widely applied for pea plants.

Since pea plants are involved in symbiotic associations with rhizobial bacteria and mycorrhizal fungi, they potentially constitute a source for addressing important questions relating to plant–microbe interactions. Previous investigations based on the analysis of pea mutants impaired in symbiosis development have yielded new insights into the role of individual genes regulating this process in legume plants, and the ongoing rapid development of methods for large-scale gene expression analyses has resulted in the identification of multiple genes, the expressions of which are specific for, or substantially enhanced by, symbiosis. Accordingly, analysis of these genes in transgenic plants may considerably enhance our understanding of the regulatory mechanisms controlling symbiosis. Currently, pea is again becoming an object of increasing research activity, and thus there is heightened demand for an efficient transformation protocol for this plant.

Although a few attempts using *Agrobacterium tumefaciens* have succeeded in transforming pea plants*,* these approaches still require further improvement due to the duration of these procedures, low efficiency, and the frequent fertility of transformed plants (De Kathen, 1990; Puonti-Kaerlas, Eriksson & Engström, 1990; Schroeder et al., 1993). In order to address these problems, one potential approach is the development of hairy root transformation procedures using *Agrobacterium rhizogenes* to generate composite plants that are characterized by transformed roots but unaltered shoots (Jensen et al., 1986; Hansen et al., 1989). This type of transformation is particularly useful for generating genetically transformed roots in a short period of time and for studying processes involved in the development of symbiotic structures on roots. The approaches used for this type of transformation are mainly based on the application of *A. rhizogenes* to wounded epicotyls or hypocotyls, with subsequent co-cultivation with bacteria.

Indeed, there have been a few previous studies in which the successful production of hairy roots in composite pea plants has been reported (Hohnjec et al., 2003; Clemow et al., 2011); however, in all cases, the most critical step is the efficiency of *Agrobacterium* infection resulting in callus formation and the subsequent generation of transformed roots. In this study, we describe a convenient protocol for highly efficient *A. rhizogenes*-mediated transformation of *P. sativum*. The composite pea plants generated using this procedure are characterized by transformed roots that are morphologically similar to those of normal roots and can be successfully nodulated by the bacterium *Rhizobium leguminosarum* bv. *viciae* or infected by the fungus *Rhizophagus irregularis*.

## Materials & Methods

### Plant material and bacterial strains

Surface-sterilized seeds of the *Pisum sativum* cultivars Finale and Frisson were treated with concentrated sulfuric acid for 5 min, washed five times with a large volume of sterile water, and then germinated on agar plates in the dark at 21–23 °C. After germination for 4 days, the seedlings were transferred to the light and placed into sterile plastic containers filled with Jensen medium and subsequently grown for further 3–4 days in a growth chamber at 21 °C and 60% humidity under 16 h/8 h light/dark cycle. The *Agrobacterium rhizogenes* strains ARqua1 (Quandt, 1993) and AR1193 (Hansen et al., 1989) and the RCAM1026 (WDCM 966) strain of *Rhizobium leguminosarum* bv. *viciae* were grown at 28 °C in Tryptone Yeast medium (Sambrook et al., 1989) containing the appropriate antibiotics.

### Transformation procedure

In order to induce the formation of hairy roots, the roots of *P. sativum* seedlings were excised in the hypocotyl region (3–4 mm below the point of cotyledon attachment). The root hypocotyls were then treated with *A. rhizogenes* ARqua 1 or AR1193 containing appropriate plasmids. For plant transformation, we used the pB7WGD plasmid carrying the *gusA*/*GFP* reporter gene construct driven by the CaMV35S promoter, which was introduced into the *A. rhizogenes* strains by electroporation. Electroporation of *A. rhizogenes* was performed using GenePulser Xcell (Bio-Rad Laboratories, Hercules, CA, USA) in 1 mm cuvette with voltage −2,500 V, capacitance 25 µF, resistance 200 ohms. The wounded root tip of the seedling was touched to the *A. rhizogenes* growing on a plate. Plants were placed on Jensen agar in plastic jars (Duchefa, Haarlem, The Netherlands), and the cut root tips were covered with wet wool and foil. The seedlings were co-cultivated with *A. rhizogenes* for 10–14 days at 21 °C (16 h/8 h light/dark) until a visible callus had developed. Emerging roots were examined using an epifluorescence stereo microscope, and transformed hairy roots were identified and selected based on *GFP* expression. Following the removal of primary roots, the seedlings were transferred to Emergence medium containing 150 mg/mL of cefotaxime and incubated for 3–4 days. Thereafter, the plants were transferred into pots of vermiculite saturated with Jensen medium containing 1.5 M M NH_4_NO_3_, grown for 2 days in a growth chamber at 21 °C and 60% humidity under a 16 h/8 h light/dark cycle, and then inoculated with *Rhizobium* strains (OD_600_ = 0.5). The pots were then placed in large plastic bags and plants were incubated under high humidity conditions for at least 1 week and then under normal conditions. The plants were watered as needed by adding the Jensen solution with 1.5 M M NH_4_NO_3_.

### RNA extraction and quantitative reverse transcription PCR (qRT-PCR)

RNA extraction was performed as described previously (Leppyanen et al., 2017). RNA (from 1 to 2.5 µg) was used for cDNA synthesis with the RevertAid Reverse Transcriptase (Thermo Scientific, Waltham, MA, USA) for 1 h at 42 °C followed by heating to 95 °C, 5 min. Aliquots of the cDNA were diluted 1:10 for qPCR analysis. qRT-PCR analysis was performed using a CFX-96 real-time PCR detection system incorporating a C1000 thermal cycler (Bio-Rad Laboratories). All reactions were done in triplicate and averaged. Cycle threshold (CT) values were obtained with the accompanying software and data were analyzed with the 2 − ΔΔ*Ct* method (Livak & Schmittgen, 2001). The relative expression was normalized against the constitutively expressed *Ubiquitin* gene. RT-PCR for *GFP* gene expression analysis was performed using Phusion Flash High-Fidelity PCR Master Mix (Thermo Fisher Scientific). All primer pairs (Table S1) were designed using Vector NTI program and produced by Evrogen (Moscow, Russia; http://www.evrogen.com).

### Mycorrhizal colonization

*Rhizophagus irregularis* (BEG144, International Bank of Glomeromycota (Dijon, France)) was kindly provided by Prof. Dirk Redecker as a soil–root-based inoculum from onion (*Allium cepa* L.) pot cultures. To obtain mycorrhizal pea plants, pea seedlings were placed in a nurse pot system with chives (*Allium schoenoprasum* L.) as nurse plants and grown in a growth chamber under conditions as previously described (Shtark et al., 2016). A mineral substrate, silica-rich marl, was used as the growth substrate. Mycorrhizal colonization was determined according to (Trouvelot, Kough & Gianinazzi-Pearson, 1986). Three parameters were considered: M%—intensity of internal colonization of the root system (reflects the proportion of the root length colonized by the fungus), A%—arbuscule abundance in mycorrhizal root fragments (characterizes the functional state of the fungus), V%—vesicle and/or spore abundance in mycorrhizal root fragments. 150 random root pieces (1 cm) were scored per replicate (not less than 750 cm/genotype).

### Histochemical staining and microscopy

The root segments and nodules were collected and stained with X-Gluc (0.2 M Na_2_PO_4_, pH7.0; 0.5 M EDTA, pH8.0; 20 mM K ferricyanide; 20 mM K ferrocyanide; 20 mM X-Gluc; 0.01% Triton X-100) for GUS staining. Tissues were incubated in staining due overnight at 37 °C. In order to assess roots mycorrhizal colonization, roots were stained with Sheaffer Black Ink at room temperature as described previously (Vierheilig et al., 1998). Photographs were obtained using an Olympus BX51 microscope (Olympus Optical Co. Europa GmbH, Hamburg, Germany) equipped with a ColorView II digital camera and analySIS FIVE analytical software (Olympus Soft Imaging Solution GmbH, Hamburg, Germany).

## Results

### Generation of pea composite plants and subsequent nodulation

In order to select for transformed hairy roots and quantify the efficiency of transformation, we introduced a plasmid carrying a fusion with the *gusA*/*GFP* reporter gene driven by the CaMV35S promoter and subsequently examined hairy roots and nodules for green fluorescent protein (GFP) or *β*-glucuronidase (GUS) staining.

**Figure 1:**
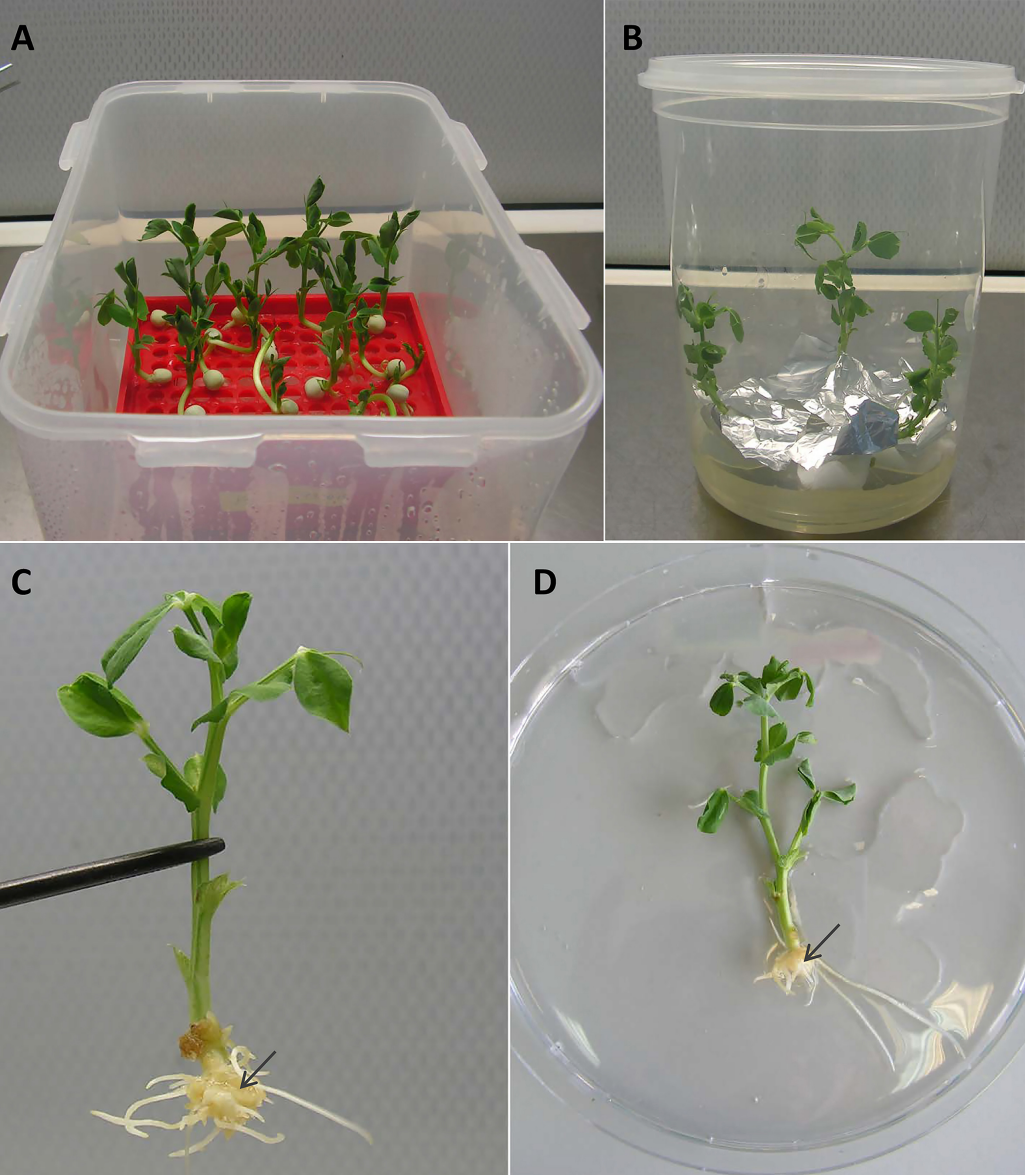
Procedure of transformation. (A) Pea seedlings were incubated in containers filled with Jensen medium placed within ventilated plastic box. (B) Incubation of pea seedlings in jars after transformation. (C) Callus formation on wounded sites of young cv. Frisson pea seedlings treated with *A. rhizogenes*. (D) General view of pea seedling after incubation on an antibiotic. The arrows indicate the callus formation.

To obtain the composite plants, young etiolated seedlings of the two cultivars Finale and Frisson were used for transformation with the ARqua1 or AR1193 strain of *Agrobacterium rhizogenes* carrying the *gusA*/*GFP* reporter construct. Using both *A. rhizogenes* strains, root transformation was induced with satisfactory efficiency, although differences were observed in number of induced roots (Table 1). Consistent with the findings of Clemow et al. (2011), we found that AR1193 was a more effective strain for hairy root transformation. In the procedure used in the present study, the plants were incubated in containers filled with Jensen medium placed within ventilated plastic box (Fig. 1A). Seedlings with one to two internodes are quite susceptible to transformation and were freshly sectioned in the hypocotyl region to remove the remainder of the root. In our transformation protocol, the wounded root tip of each seedling was touched to growing on a plate *A. rhizogenes* containing a plasmid that harbored the *gusA*/*GFP* reporter. Following transformation, the pea seedlings were incubated in jars and the root tips were maintained under high-humidity conditions (Fig. 1B).

**Table 1:** *Agrobacterium rhizogenes*-mediated transformation of *Pisum sativum* cv. Frisson using the ARqua 1 and AR1193 strains of *A. rhizogenes*.

	**Total amount of plants**	**Number of transformed composite plants**^a^	**Total number of hairy roots**	**Number of hairy roots per transformed composite plant**
*Agrobacterium rhizogenes* Arqua1	114 (10)^b^	80 (10)^b^	130	1,6
*Agrobacterium rhizogenes* AR1193	62 (5)^b^	50 (5)	88	1,8

**Notes.**

a Transformed composite plants that gave rise to at least one hairy root per plant.

b The number of independent experiments is indicated within brackets.

**Figure 2:**
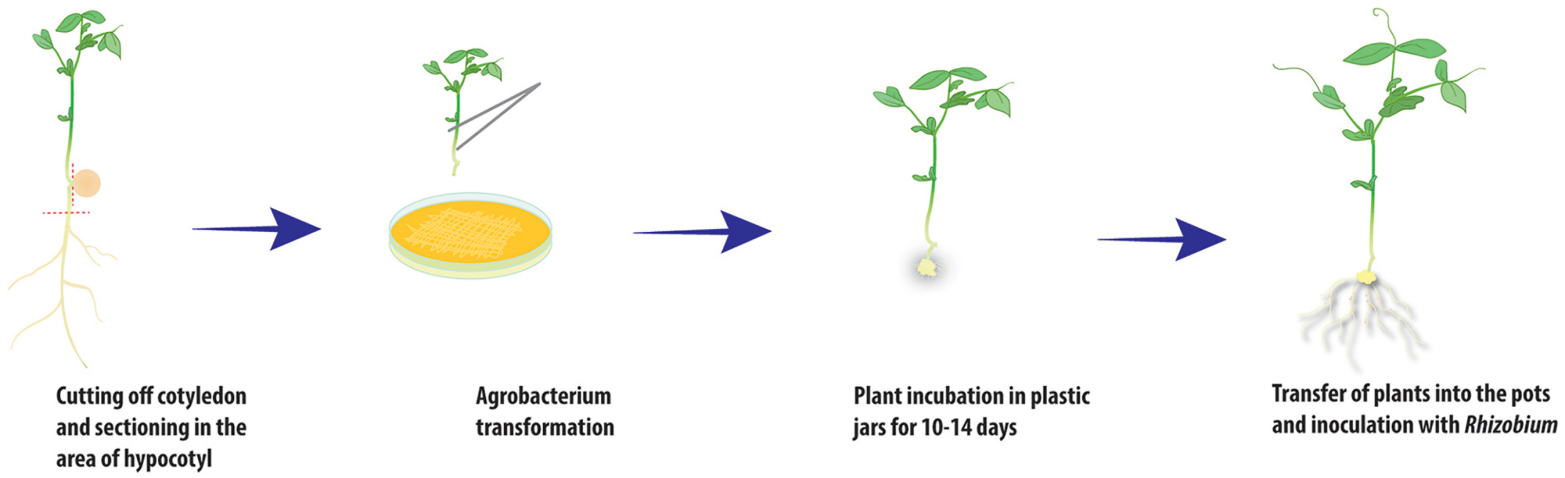
Scheme of *A. rhizogenes*-mediated transformation of pea plants.

**Figure 3:**
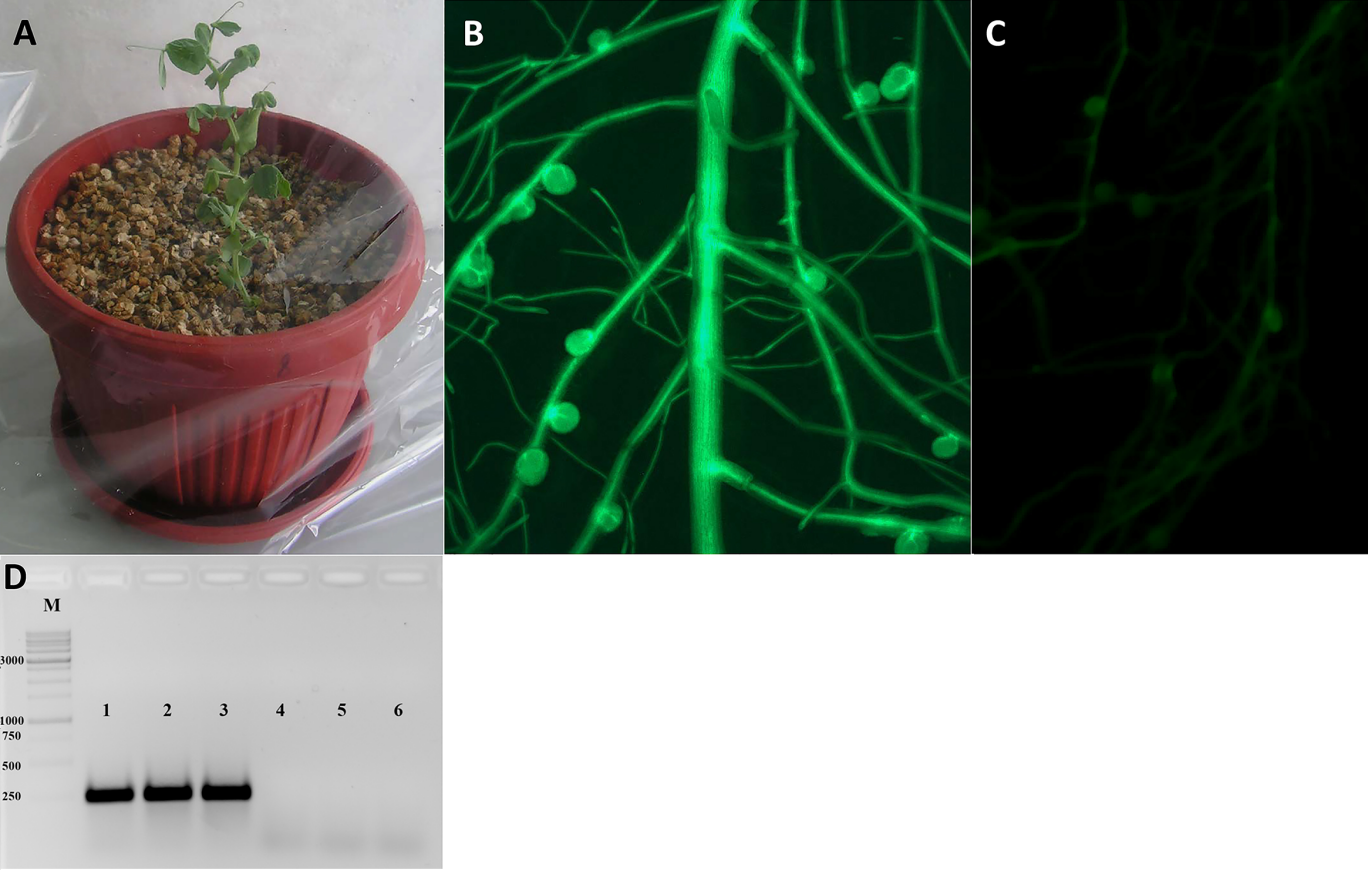
Analysis of transformed pea plants after *R. leguminosarum* inoculation. (A) Pea plants incubated under high humidity conditions following their transfer to pots. (B) The plant marker GFP was visualized in transgenic roots and nodules using an epifluorescence microscope. (C) Non-transformed roots of pea plants. (D) The expression of *GFP* gene in transgenic nodules of *P. sativum* cv. Frisson using RT-PCR (1–3), as a control the nodules of non-transformed plants were used (4–6).

After 10–14 days, calli started to develop at the wound sites in seedling of the cultivar Frisson (Fig. 1C), whereas the initiation of callus development was slightly more prolonged (16–18 days) for seedlings of the Finale cultivar. Approximately 70%–80% of the wound sites gave rise to callus formation and the subsequent development of transformed roots. At this stage, a number of primary roots appeared (Fig. 1C). These primary roots were removed prior to antibiotic treatment in order to stimulate development of the transformed roots. We found that incubation on an antibiotic-containing medium prevented excessive growth of *A. rhizogenes* and the development of root abnormalities (Fig. 1D). After incubation, the plants were subsequently transferred to pots saturated with a low-nitrate nutrient solution to prevent nitrogen starvation (Figs. 2 and 3A). Since the seedlings were cultivated under axenic conditions, they may also be used to obtain the cultivated hairy roots at this stage.

After transferring to pots and cultivating for a few days, the plants were inoculated with *R. leguminosarum* bv. *viciae* RIAM1026 and we observed that root nodules generally appeared after 2–3 weeks. In order to maintain high humidity conditions after transfer, we placed the pots into large plastic packages in which they were retained for at least 1 week, because it was easy to ventilate the plants during cultivation (Fig. 3A). After 2–3 weeks, we were able to visualize the emergence of nodules on transformed roots using an epifluorescence stereo microscope (Fig. 3B). We found that the efficiency of nodule formation varied depending on the cultivar used, with cv. Finale showing a high level of nodule formation, whereas comparatively fewer nodules developed on the transformed roots of cv. Frisson plants. GFP staining was used as a simple marker to score for transformed roots 3 weeks after infection with *R. leguminosarum*. We found that approximately 70% of the inoculated plants had at least one transformed root, and that composite plants had an average of two to three such roots, thereby indicating the high frequency of transformation achieved using this protocol, which will greatly facilitate studies involving gene function analysis.

### Studying the possibility of mycorrhization for transformed roots in pea

In the present study, we also developed approaches for inoculation of transgenic hairy roots with the AM fungus *R. irregularis* using a nurse-plant system. As nurse plants, we used four young onion seedlings grown in pots containing Hoagland medium-saturated substrate for 4 weeks. After cultivation on antibiotic-containing medium, the transformed pea seedlings were transferred to the pots containing these nurse plants.

**Figure 4:**
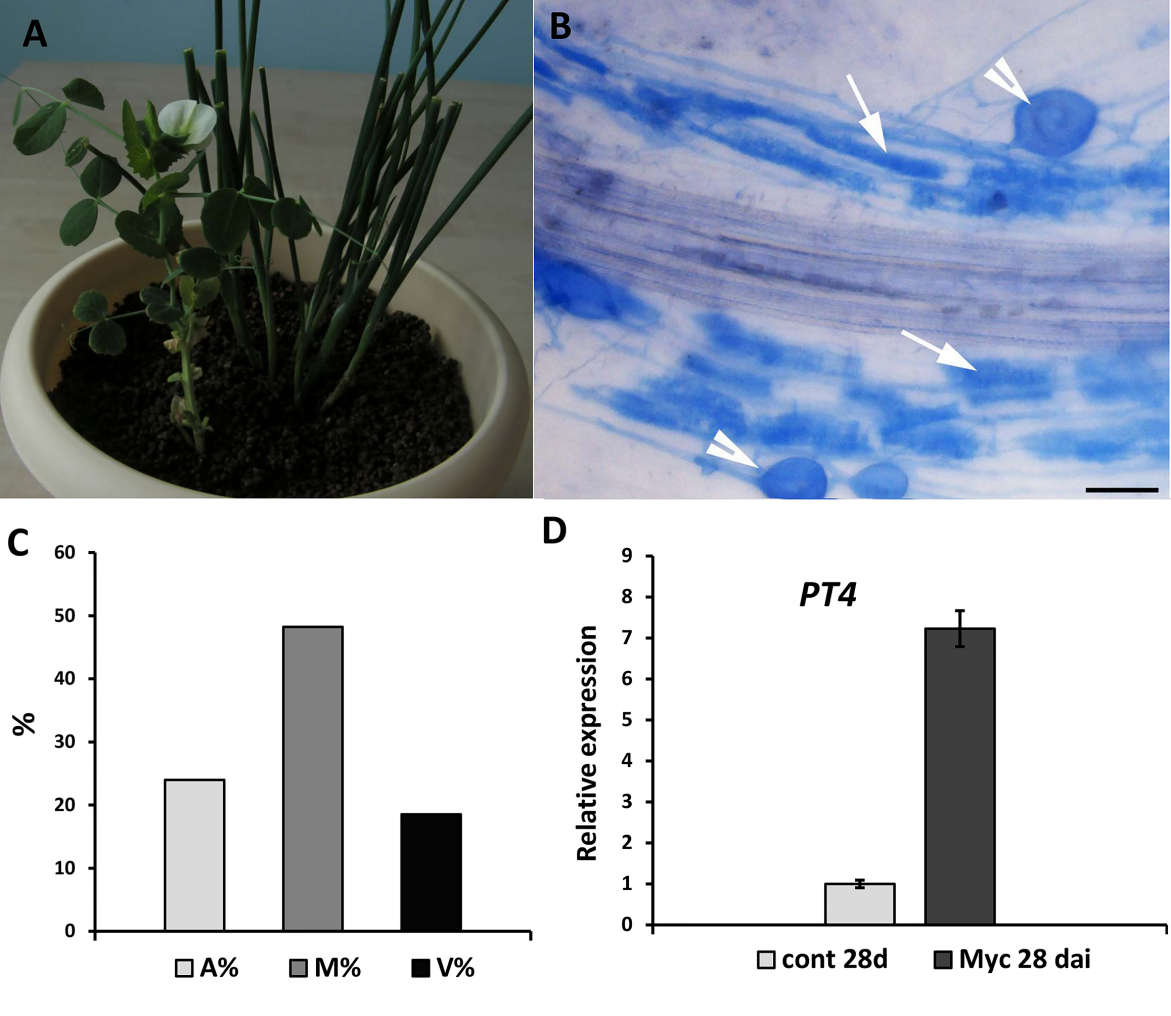
Inoculation of pea plants with the AM fungus *R. irregularis* using a nurse-plant system. (A) General view of pea plants in a nurse-plant system during mycorrhizal colonization. (B) Arbuscular mycorrhizal fungi colonize efficiently *GUS*-transformed pea roots in a nurse plant system. White arrows indicate cells with arbuscules, white arrowheads point at vesicles. Scale bars: *a* = 50 µm. (C) Three parameters were considered: M%, intensity of internal colonization of the root system, A%, arbuscule abundance in mycorrhizal root fragments and V%, vesicle and/or spore abundance in mycorrhizal root fragments. (D) Expression of *PT4* gene encoding mycorrhiza-inducible inorganic phosphate transporter in non-inoculated (control) and inoculated (Myc) transgenic roots (28 days after inoculation). mRNA levels were normalized against *Ubiquitin*, and values were calculated as ratios relative to non-inoculated root expression level.

In order to maintain the high humidity conditions after transfer, we placed the pots into large plastic packages for at least 1 week, similar to the procedure used for rhizobial inoculation (Fig. 4A). After 4 weeks of growth, we assessed the efficiency of symbiosis development with AM fungi and detected highly effective inoculation (Figs. 4B and 4C). Expression levels of marker typically associated with AM fungus symbiosis development, namely, the *PT4* gene encoding the mycorrhiza-inducible inorganic phosphate transporter, confirmed induction of the symbiotic association (Fig. 4D). These findings indicate that the protocol we describe here may be useful for the analysis of another type of symbiosis in composite pea plants with transformed roots.

## Discussion

In recent years multiple studies have led to the development of sufficiently effective approaches for pea transformation using *A. tumefaciens* (Bean et al., 1997; Polowick, Quandt & Mahon, 2000; Wisniewski & Brewin, 2000; Schneider et al., 2002; Pniewski & Kapusta, 2005; Svabova, Smykal & Griga, 2005; Krejčí et al., 2007; Sehgal, Bhat & Raina, 2008; Aftabi, Teressa Negawo & Hassan, 2017). However, due to the duration of these procedures, the alternative hairy root transformation approaches using *A. rhizogenes* require further development.

*A. rhizogenes-* mediated transformation has previously been described for a few legume species, initially being applied for the expression of soybean genes in *Lotus corniculatus* (Jensen et al., 1986; Jørgensen et al., 1988; Hansen et al., 1989). Subsequently, approaches were described for transformation and regeneration of the model legume plant *Lotus japonicus* (Handberg & Stougaard, 1992; Stiller et al., 1997). Hairy root transformation was also developed for another model legume, *Medicago truncatula* (Boisson-Dernier et al., 2001; Gourion et al., 2013), and protocols for hairy root transformation have been adapted for other legume plants, including *Vigna aconitifolia* (Lee et al., 1993), *Trifolium repens* and *Trifolium pratense* (Díaz et al., 1989; Díaz, Spaink & Kijne, 2000), *Glycine max* (Cheon et al., 1993), *Vicia hirsute* (Quandt, 1993)*, Phaseolus vulgaris* L. (Estrada-Navarrete et al., 2006) and peanut *Arachis hypogea* (Sinharoy et al., 2009). Furthermore, previous studies have also reported the successful production of hairy roots in composite pea plants (Hohnjec et al., 2003; Clemow et al., 2011).

In an effort to develop approaches for highly efficient *A. rhizogenes-* mediated hairy root transformation in pea, we have previously treated the needle-wounded hypocotyls of peas with an inoculum of *A. rhizogenes* (Osipova et al., 2012) as it was described (Hohnjec et al., 2003). However, although transformed pea roots and nodules were obtained and enabled us to investigate certain pea transgenes, this approach did not result in highly efficient transformation. Accordingly, in the present study, we sought to enhance the efficiency of transformation by inducing callus formation on the wounded sites of freshly sectioned hypocotyls of pea seedling via incubation with *A. rhizogenes*.

Using our novel methodology for hairy root induction in pea, we demonstrate the successful transformation of *P. sativum* by the ARqua 1 and AR1193 strains of *A. rhizogenes*, whereby transgenic roots in the composite plants showed expression of the genes encoding the green fluorescent protein and GUS under the control of the CaMV35S promoter. Subsequent to infection with *A. rhizogenes*, approximately 70% to 80% of pea seedlings developed transgenic hairy roots. Since the seedlings were cultivated under axenic conditions, they may also be used to obtain the cultivated hairy roots.

We observed that these transgenic roots can be efficiently nodulated by the rhizobial bacterium *R. leguminosarum* and infected by the AM fungus *R. irregularis*. Morphologically, the nodules that developed on transgenic roots were found to be identical to those characterizing wild-type roots. We also demonstrate that a supply of nitrogen is important for enhancing the growth of transformed plants following their transfer from rich media to pots. Furthermore, we also verified the development of a symbiosis with AM fungi via the effective induction of markers typically associated with this process. We thus demonstrate an approach for the comparatively rapid generation of symbiotic structures on transgenic pea roots, and accordingly consider that our protocol can be employed as a rapid and efficient tool for studying the genes involved in plant–microbe interactions in *P. sativum*.

## Conclusions

A convenient protocol for highly efficient *A. rhizogenes*-mediated transformation of pea *P. sativum* has been developed. In pea composite plants, the hairy roots can be nodulated successfully by *R. leguminosarum* bv. *viciae* bacteria or infected by *R. irregularis* fungi.

##  Supplemental Information

10.7717/peerj.6552/supp-1 Supplemental Information 1 Raw data

10.7717/peerj.6552/supp-2 Table S1 List of primers used for PCR

10.7717/peerj.6552/supp-3 Figure S1 Map of binary vector used for pea transformation

## Additional Information and Declarations

### Data Availability

The following information was supplied regarding data availability: The raw data are available in Supplementary File 1.
