# Supplementary material for: Agrobacterium rhizogenes—mediated transformation of Pisum sativum L. roots as a tool for studying the mycorrhizal and root nodule symbioses
Source: PeerJ. 2019 Mar 6;7:e6552. doi: 10.7717/peerj.6552 (PMC6408910; doi:10.7717/peerj.6552)
Supplement: Supplemental Information 1 [file peerj-07-6552-s001.pdf]

Raw data

Table 1. ***Agrobacterium rhizogenes*-mediated transformation of *Pisum sativum* cv. Frisson**

	**Total amount of plants**	**Number of transformed composite plants***	**Total number of hairy roots**	**Number of hairy roots per transformed composite plant**
Agrobacterium rhizogenes Arqua1	114 (10) **	80 (10)**	130	1,6
Agrobacterium rhizogenes AR1193	62 (5)**	50 (5)	88	1,8

*Transformed composite plants that gave rise to at least one hairy root per plant.

**The number of independent experiments is indicated within brackets
