# Supplementary material for: Agrobacterium rhizogenes—mediated transformation of Pisum sativum L. roots as a tool for studying the mycorrhizal and root nodule symbioses
Source: PeerJ. 2019 Mar 6;7:e6552. doi: 10.7717/peerj.6552 (PMC6408910; doi:10.7717/peerj.6552)
Supplement: Table S1 [file peerj-07-6552-s002.pdf]

Table S1. List of primers used for PCR.

Gene name	Forward primer	Reverse primer
Ubiquitin	5'-ATGCAGATC/TTTTGTGAAGAC-3'	5'-ACCACCACGG/AAGACGGAG-3'
PT4	5'-CATTCTGAGCTGATCGTCGTATGGTG-3'	5'-GCATTAAGAATTTCCCCAGAGGTC-3'
GFP	5'-ATCGACTTCAAGGAGGACGG-3'	5'-CTTTGCTCAGGGCGGACTGG-3'
