# Supplementary figures and images for: Agrobacterium rhizogenes—mediated transformation of Pisum sativum L. roots as a tool for studying the mycorrhizal and root nodule symbioses
Source: PeerJ. 2019 Mar 6;7:e6552. doi: 10.7717/peerj.6552 (PMC6408910; doi:10.7717/peerj.6552)

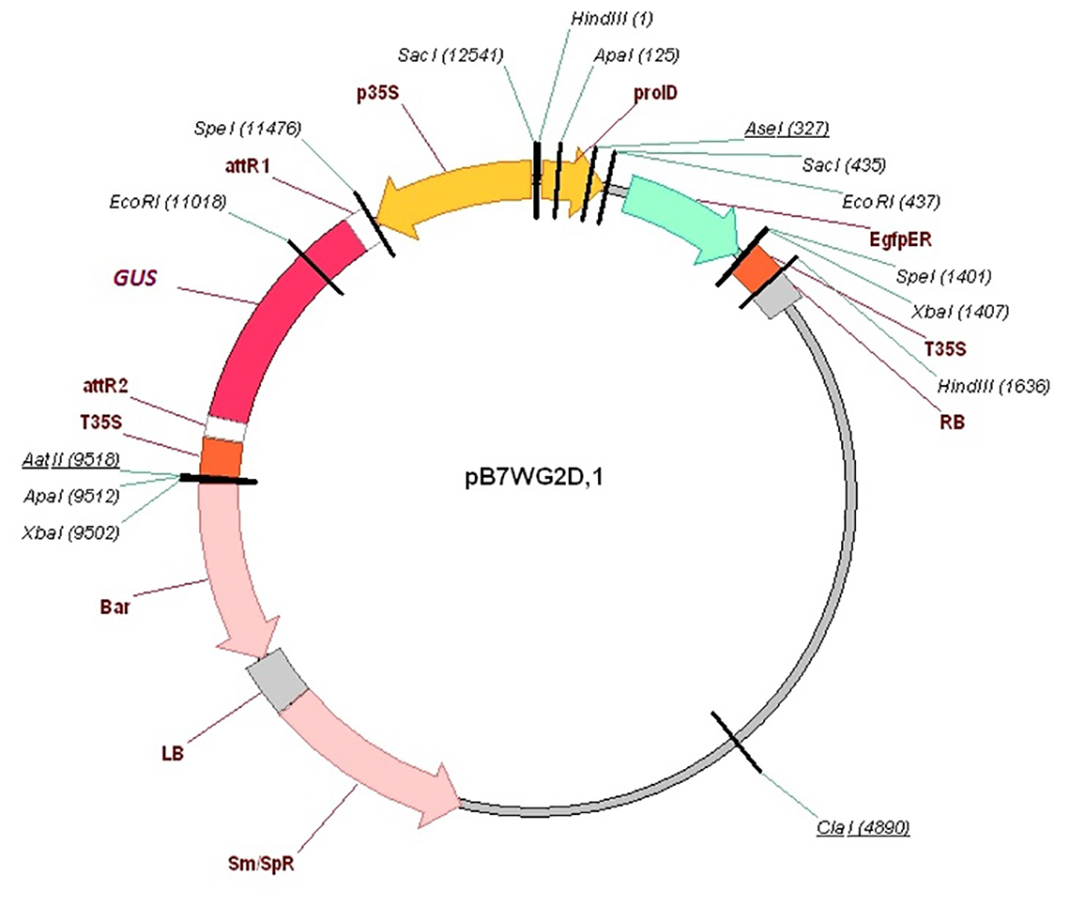

Supplement: Figure S1 [file peerj-07-6552-s003.jpg]
